# Numerical Analysis and Design of Active Waveguide Bragg Gratings for High-Contrast Thermal Sensing and Tunable Linear Thermal Gradient Threshold Detection

**DOI:** 10.3390/s26175495

**Published:** 2026-08-30

**Authors:** Ángel Sanz-Felipe, Juan Antonio Vallés

**Affiliations:** 1 Applied Physics Department, Faculty of Science, University of Zaragoza, 50009 Zaragoza, Spain; juanval@unizar.es; 2 Engineering Research Institute of Aragon (I3A), University of Zaragoza, 50018 Zaragoza, Spain

**Keywords:** active waveguide, integrated Bragg gratings, waveguide monolithic lasers, transfer matrix method, thermal gradient sensors, optical switches

## Abstract

Active waveguide Bragg gratings (AWBGs) combine the narrowband reflectivity of Bragg gratings with the optical gain of rare-earth-doped media, representing a versatile platform for monolithic lasers and amplifying reflectors. The lasing regime in these photonic structures is governed by specific design and pumping parameters, exhibiting critical thresholds below which laser emission is suppressed. This work separately explores the potential of AWBGs as high-contrast thermal sensors and threshold-triggering switches. The AWBG thermal response is numerically analyzed by means of the Transfer Matrix Method. Under uniform heating, the Bragg wavelength shift shows sensitivities of 12 pm/°C. The resulting laser peak, with only 1 nm bandwidth and several orders of magnitude above the noise floor, significantly improves the peak-to-background contrast of equivalent passive designs. Furthermore, the impact of linear longitudinal thermal gradients is evaluated. The thermal chirp induced reduces the grating’s efficiency, potentially quenching the laser emission if the system is biased near its operating threshold. Our numerical results indicate that AWBGs can function as threshold-triggering thermal gradient detectors where the detection threshold can be adequately chosen at the design stage for a detection range up to approximately 12 °C/mm, and selectively tuned through pump power regulation once fabricated. These findings, obtained under the numerical approximations considered, position AWBGs as a promising solution for advanced thermal monitoring and optical safeguarding applications, pending experimental validation.

## 1. Introduction

Active waveguide Bragg gratings (AWBGs) consist of periodic refractive index perturbations inscribed into rare-earth-doped waveguides. This architecture successfully combines the efficient narrowband reflectivity of Bragg gratings (BGs) [1] with the optical gain of an active medium [2,3]. Under proper design and operating conditions, these devices operate as laser cavities or optical amplifiers [4], making them versatile for monolithic lasers and amplifying reflectors [5,6,7,8]. The transition into the lasing regime is governed by a delicate balance between structural parameters (grating length, efficiency, and intrinsic material attenuation) and operational variables, such as pump power [9].

Over the last few decades, passive BGs have attracted significant interest for sensing applications due to their high sensitivity to physical magnitudes such as strain, pressure, temperature, external refractive index, humidity and magnetic fields [10,11,12,13,14,15,16,17], with many simultaneous multi-parameter sensing studies. Their immunity to electromagnetic interference, small footprint, and cost-effectiveness have established them as a mature sensing alternative, even in extreme environments [18,19]. Variations in the target physical magnitude typically induce a spectral shift in the BG reflectivity, a property widely exploited for high-precision monitoring [20]. For thermal sensing, this mechanism is dominated by the thermo-optic effect and thermal expansion, which modify the effective refractive index and the grating period, respectively. Despite their success, passive designs often suffer from limited signal-to-background contrast and relatively broad reflection bandwidths. These limitations have been addressed via paired-BG designs [21], or bypassed entirely through the generation of laser lines when an active medium is incorporated [22,23].

Utilizing AWBGs for sensing may address these limitations while expanding device functionalities. First, AWBGs can generate an ultra-narrow monochromatic laser peak standing several orders of magnitude above the noise floor in a compact structure [5]. Second, besides the Bragg peak shift, spatial variations in the sensing magnitude—such as sharp thermal gradients—can introduce a phase chirp along the grating. This distortion alters the cavity’s spectral response by broadening the resonance peak, which severely degrades efficiency [24], potentially dropping the system below its threshold and abruptly quenching the laser emission. Research into thermal gradient sensing is of utmost importance, as localized temperature variations govern critical processes in aerospace systems [25], battery packs [26,27], biomedical applications [28], and high-power electronic devices [29]. These two distinct operating modes enable AWBGs to operate, separately, as high-contrast thermal sensors and as threshold-triggering optical switches.

To fully exploit these advanced structures, developing robust predictive numerical frameworks is essential prior to physical manufacture. In typical fabrication setups—such as femtosecond laser inscription—achieving absolute control over structural parameters (losses, index modulation profile, and final coupling) remains a technological challenge. Given the high sensitivity of AWBGs to these parameters, even minor fabrication tolerances can drastically alter the device functionality laser threshold conditions. In this context, simulations based on the Transfer Matrix Method (TMM) are widely used to predict passive BG optical behavior [30,31], with extended TMM-based models yielding closer experimental-to-theoretical agreement [32]. However, while numerical methods for active media exist [33], incorporating rare-earth contributions into optical propagation calculations significantly increases computational complexity and time.

In this work, we present a numerical design framework for Er/Yb-codoped AWBGs that couples the TMM with an active-medium power propagation solver, explicitly distinguishing two operational modes of the proposed platform: (i) high-contrast uniform thermal sensing, and (ii) a threshold-triggered “on/off” thermal gradient switch. Remarkably, unlike passive devices whose response is fixed upon fabrication, we show that the AWBG gradient detection threshold can be dynamically post-tuned by simply regulating the optical pump power, offering a versatile safeguarding solution for integrated photonics. This numerical study offers a potential predictive design tool to optimize AWBG reliability prior to physical fabrication. The work is organized as follows. Section 2 describes the numerical methods employed to simulate uniform BGs under thermal variations and power propagation in the active medium. Section 3 presents the simulation results and discussions. Section 4 summarizes the principal findings and conclusions.

## 2. Materials and Methods

The numerical modeling of the proposed AWBG is grounded in evaluating the vulnerability of its spectral response to temperature variations. In order to take advantage of the rare-earth amplification, the uniform AWBG design is focused on the Bragg condition, $\lambda_{B}$, defined as:

(1) $$\lambda_{B}=2n\Lambda$$

where Λ is the grating period, and the effective refractive index of the waveguide after the core inscription process is denoted as n for simplicity. Both parameters are intrinsically temperature-sensitive, so any thermal perturbation directly distorts this resonance condition. To numerically evaluate these phenomena, the passive uniform BG is first modeled using the TMM with MATLAB (v2025), and the resulting spectral variations are subsequently integrated into our experimentally validated active-medium propagation algorithm to evaluate the effects on the AWBG performance.

### 2.1. Thermal Dependencies in the Transfer Matrix Method

The TMM is a widely established numerical tool for analyzing light propagation in perturbed or weakly coupled waveguides by implementing the analytical solutions of the Coupled Mode Theory (CMT) equations [1]. In this approach, the BG of length L is divided into N uniform segments of length Δz=L/N. The co- and counter-propagating field amplitudes (A and B respectively) at the i-th segment’s input and output are related via its local transfer matrix, $T_{i}$, as follows:

(2) $$\left(\begin{array}{c} A_{i}\\ B_{i} \end{array}\right)=T_{i}\left(\begin{array}{c} A_{i-1}\\ B_{i-1} \end{array}\right)$$

where the matrix elements are derived from the CMT boundary conditions:

(3) $$\begin{array}{c} T_{11}=T_{22}^{*}=\cosh\left(\gamma \Delta z\right)+i\frac{\delta }{\gamma }\sinh\left(\gamma \Delta z\right)\\ T_{12}=T_{21}^{*}=i\frac{\kappa }{\gamma }\sinh\left(\gamma \Delta z\right) \end{array}$$

Here, κ is the BG coupling coefficient which dictates grating efficiency, δ is the detuning number from resonance, and $\gamma =\sqrt{\kappa^{2}-\delta^{2}}$. These parameters are governed by the physical properties of the guiding material and the fabrication parameters:

(4) $$\kappa =\frac{\pi \Delta n\eta }{\lambda },\;\delta =2\pi n\left(\frac{1}{\lambda }-\frac{1}{\lambda_{B}}\right)$$

where Δn is the effective refractive index modulation amplitude, and η is the modal overlap factor which describes the fraction of the guided mode field power within the index perturbation. For a rectangular grating profile with a peak index variation $\Delta n_{o}$ and a duty cycle DC, the effective index modulation is given by $\Delta n=2\Delta n_{o}DC\mathrm{sinc}\left(\pi DC\right)$ [34]. The average index increase, $\bar{\Delta n}=\Delta n_{o}DC$, induces a slight variation in the background effective index, and consequently shifts the Bragg condition from the $\lambda_{D}$ nominal design value [1]: $\lambda_{B}=\left(1+\eta \frac{\bar{\Delta n}}{n}\right)\lambda_{D}$.

The complex field transmission and reflection coefficients for each segment and wavelength are determined by the transfer matrix elements: $t=1/T_{22}$, $r=T_{12}/T_{22}$. Analogously, the global BG coefficients are obtained through the cumulative transfer matrix, $T=T_{N}T_{N-1}\ldots T_{2}T_{1}$. In a uniform BG, at the Bragg wavelength (δ=0), the maximum power reflectivity is given as a function of the coupling coefficient and the grating length:

(5) $$R_{max}=\tanh^{2}\left(\kappa L\right)$$

In this expression, propagation losses have been neglected for simplicity (they are integrated into the active behavior calculation in Section 2.2).

Given the Bragg condition, thermal effects influence the model primarily by altering the effective refractive index and the grating period (and indirectly through the Bragg wavelength). Thus, the TMM parameters become temperature-dependent functions.

#### 2.1.1. Uniform Heating

A uniform temperature change primarily induces a shift in the Bragg wavelength derived from Equation (1):

(6) $$\frac{d\lambda_{B}}{dT}=2\left(\Lambda \frac{\partial n}{\partial T}+n\frac{\partial \Lambda }{\partial T}\right)=\lambda_{B}\left(\alpha_{n}+\alpha_{\Lambda }\right)$$

where $\alpha_{n}=\frac{1}{n}\frac{\partial n}{\partial T}$ is the normalized thermo-optic coefficient, and $\alpha_{\Lambda }=\frac{1}{\Lambda }\frac{\partial \Lambda }{\partial T}$ is the thermal expansion coefficient. Assuming the thermal coefficients to be constant for moderate temperature variations, this relationship yields a linear spectral shift of the Bragg peak, proportional to the sum of both coefficients:

(7) $$\Delta \lambda_{B}=\lambda_{B}\left(\alpha_{n}+\alpha_{\Lambda }\right)\Delta T$$

The thermal shift sensitivity is then evaluated as the ratio of the Bragg wavelength drift to the applied temperature change:

(8) $$S_{T}=\frac{\Delta \lambda_{B}}{\Delta T}=\lambda_{B}(\alpha_{n}+\alpha_{\Lambda })$$

Similarly, the thermal variation of the coupling coefficient at the resonance peak can be obtained from Equation (4):

(9) $$\begin{array}{c} \frac{d\kappa }{dT}=\frac{\pi \eta }{\lambda_{B}}\frac{\partial \left(\Delta n\right)}{\partial T}+\frac{\pi \Delta n}{\lambda_{B}}\frac{\partial \eta }{\partial T}-\frac{\pi \eta \Delta n}{\lambda_{B}^{2}}\frac{\partial \lambda_{B}}{\partial T}=\\ \;=\kappa \alpha_{n}+\kappa \frac{2}{V^{2}-1}\left(\alpha_{n}+\alpha_{\Lambda }\right)-\kappa \left(\alpha_{n}+\alpha_{\Lambda }\right)=\frac{\left(2\alpha_{n}+\left(3-V^{2}\right)\alpha_{\Lambda }\right)}{V^{2}-1}\kappa \end{array}$$

In this expression, the thermal variation of η is governed by the waveguide geometry, operation wavelength, and the core/cladding refractive indices through the waveguide V-parameter ($V=\frac{2\pi a}{\lambda }\sqrt{n_{core}^{2}-n_{clad}^{2}}$, where a is the core radius), and the approximation to the Marcuse formula, $\eta \approx 1-1/V^{2}$, has been used in the derivation for simplicity (detailed derivative steps have been omitted for brevity). Also, the core, cladding and refractive index modulation have been assumed to share the same thermo-optic coefficients, as a first approximation. Considering the typical single-mode value V≈ 2 (in our reference sample, V≈ 1.8), all the terms in the final expression are proportional to the same order of thermal coefficients, which typically range between 10^−5^ and 10^−6^ °C^−1^ for the substrates of interest. So, moderate heating (ΔT< 100 °C) induces a relative change in κ of less than 0.1%. Since this perturbation only affects the fourth significant digit of the coupling strength, the grating efficiency remains practically unaltered. As a result, the thermal response manifests solely as a spectral shift of the reflection peak.

#### 2.1.2. Linear Longitudinal Thermal Gradient

While uniform heating only translates the reflection spectrum without altering its shape, a linear thermal profile introduces a spatial dependence along the grating’s longitudinal axis, inducing a chirp effect in the detuning: $\delta \left(z\right)=\delta_{o}+\phi z$. Consequently, the detuning number, which measures the deviation from the local Bragg condition, becomes position-dependent. This thermally induced chirp ϕ can be derived from the temperature dependence of the detuning and the local thermal gradient:

(10) $$\phi =\frac{\partial \delta }{\partial z}=\frac{\partial \delta }{\partial T}\frac{\partial T}{\partial z}=\frac{\partial \delta }{\partial T}\nabla T$$

The derivative of the detuning number with respect to temperature can be obtained from its fundamental definition, Equation (4), through the thermal coefficients:

(11) $$\frac{\partial \delta }{\partial T}=2\pi \left(\frac{1}{\lambda }-\frac{1}{\lambda_{B}}\right)\frac{\partial n}{\partial T}+\frac{2\pi n}{\lambda_{B}^{2}}\;\frac{d\lambda_{B}}{dT}=\delta_{o}\alpha_{n}+\frac{2\pi n}{\lambda_{B}}\left(\alpha_{n}+\alpha_{\Lambda }\right)$$

in which the result of Equation (6) has been used to reach a final expression. For wavelengths in close proximity to the resonance, the $\delta_{o}\alpha_{n}$ baseline detuning term is negligible (roughly three orders of magnitude lower) compared to the second term in the right-hand side, so the main contribution to this variation can be assumed again to be proportional to the sum of the thermal coefficients.

Through this mathematical framework, applying a longitudinal thermal gradient alters the grating performance: each grating segment experiences a locally distinct Bragg condition, introducing a z-dependent detuning. As a consequence, waves reflected at different longitudinal positions accumulate a progressive phase mismatch rather than adding up in phase. This spatial variation compromises the phase coherence of the backward-propagating waves, diminishing constructive interference and leading to a severe degradation of the overall grating efficiency. In addition, a shift in the reflection spectrum also occurs, as every point along the grating defines a local Bragg condition. Specifically, under a linear gradient, the central position of the cumulative spectrum aligns with the wavelength corresponding to the net shift induced by half the temperature difference between the grating ends, as described by Equation (7).

The possible z-dependence of the modal-overlap factor due to the thermal chirp has been neglected: it would contribute to an apodization, $\kappa \left(z\right)$, but its magnitude remains bounded by the thermal coefficients already discussed for Equation (9), and is therefore negligible compared to the dominant phase chirp considered here.

### 2.2. Optical Power Propagation in AWBGs

Although the TMM is widely used for modeling passive designs, it fails to account for the active behavior: the optical gain is highly non-linear and spatially dependent, as it relies directly on the local population densities of the rare-earth ionic energy levels, which are governed by the position-dependent pump and signal intensities. To address this, a numerical algorithm previously developed and experimentally verified [5,9] is employed to simulate the optical power propagation through an Er/Yb-codoped integrated AWBG. A Runge–Kutta iterative calculation is employed to determine the co- and counter-propagating powers (subscripts R and L respectively) and their derivatives along the waveguide propagation direction z in each Δz grating segment:

(12) $$\frac{dP_{R/L}}{dz}=\left(A\left(z\right)-\beta +\frac{\ln\left(t\right)}{\Delta z}\right)P_{R/L}-\frac{\ln\left(t\right)}{\Delta z}P_{L/R}$$

Here, the first term on the right determines the changes in the power propagating in the calculation direction, considering the Er^3+^/Yb^3+^ amplification, $A\left(z\right)$, the medium’s propagation loss, β, and the Bragg grating effect by means of the Δz segment’s transmission coefficient t, which integrates the passive TMM results and thermal dependencies into the active algorithm ($R=1-t^{N})$, directly connected to the grating coupling, as derived from Equation (5). The second term represents the cross-talk between both propagation directions: the fraction of reflected power in each segment contributes to increasing the power propagating in the opposite direction.

To trigger lasing action, a symmetric bidirectional pump power (λ= 976 nm) is injected at both grating ends. This pump will be partially absorbed and converted into fluorescence by the Er/Yb ions. As this fluorescence propagates along the medium, it undergoes optical amplification to produce amplified spontaneous emission (ASE), peaking at the Er^3+^ ion main amplification wavelength (λ= 1534 nm) for which the BG is designed. The mutual dependence between $P_{R}$ and $P_{L}$ in Equation (12) may lead to a self-amplified multiple reflection behavior that, ultimately, induces a laser cavity mechanism if the proper conditions are matched. An iterative calculation is then run until a convergent solution is achieved.

In order to simulate the active behavior of the device, the spectral response of BGs must be integrated to the active propagation solver. Given the highly monochromatic nature of these optimized cavities, as experimentally observed in our previous work [5], a delta-function reflection spectrum was successfully employed [5,9], which evaluated the active response within the 1525–1540 nm range with a 1 nm resolution (more details of the method, dopant parameters and convergence analysis can be found in those works). As described in the previous sections, under thermal variations, the grating spectrum experiences shifts and—in the thermal gradient case—broadening. Therefore, analyzing these spectral changes within our thermal ranges is essential to verify whether the active device remains operating within the rare-earth amplification band. It should be noted that, throughout Section 3.2, the lasing-threshold dataset (ASE power and critical coupling coefficient as a function of grating length, pump power, and losses) is reused from our previous work [9], generated under equivalent, unperturbed conditions. Some additional control simulations have been deployed to verify the physical validity of this approach, as it will be justified and discussed in Section 3.

### 2.3. Physical Principles of Sensing Mechanisms and Numerical Workflow

Two distinct operational sensing mechanisms under thermal variations in the functional glass host are considered in this work:

- Uniform heating spectral shift mode (Section 2.1.1): The passive BG response shifts without efficiency degrading; new active simulations are unnecessary here, only those to verify that the Bragg peak remains within the Er/Yb gain bandwidth. This is analyzed in Section 3.1.
- Thermal gradient switching mode (Section 2.1.2 and Section 2.2): A longitudinal thermal gradient degrades the passive BG efficiency until the active cavity falls below the lasing condition, abruptly quenching stimulated emission to low-power ASE, enabling a dynamic “on/off” thermal gradient detector. This is analyzed in Section 3.2.

## 3. Results and Discussion

The aim of this work is to analyze the laser performance of AWBGs under thermal variations by combining the thermally dependent TMM framework with active laser response simulations. To ensure physical grounding, all numerical simulations adopt the baseline structural and spectroscopic parameters summarized in Table 1 (unless stated otherwise), which correspond to an experimental Er/Yb-codoped AWBG sample fabricated and analyzed at room temperature (RT) in our previous works [5,9]. Although the present study is strictly theoretical and numerical, the adoption of this previously reported experimental parameters serves as a physically realistic framework.

The bulk material of this reference AWBG consists of a compositionally tuned phosphate-based glass substrate [35], in which the BG was inscribed via femtosecond laser process, after duty cycle (DC) optimization to achieve highly efficient reflective gratings [31] prior to active purposes. The shift from the $\lambda_{D}$ nominal design Bragg wavelength is negligible with these parameters (only 0.03 nm). The modal overlap factor, η, is varied in the simulations to adjust the grating efficiency. While in fabrication this tuning is typically achieved by adjusting the peak index variation, $\Delta n_{o}$, the overlap factor is highly sensitive and difficult to control experimentally [31]. Therefore, we employ η as an adjustable simulation parameter.

Thermal coefficients of the reference sample could not be directly measured, so literature-based values matching our experimental glass stoichiometry (e.g., SCHOTT LG-750 and LG-940 [36]) are assumed. As a first approach, the thermal coefficients are assumed constant within the moderate thermal variations studied (<100 °C). The propagation loss coefficient considered, β, falls within the range for this type of material [35] and matches the value observed in our previous experimental-to-theoretical comparison [5]. These approximations provide a reliable baseline for the numerical analysis of these devices.

The thermal dependencies of passive BG spectral responses are first simulated to analyze the baseline structural response, prior to integrating it into active dynamics. These simulations are based strictly on the TMM described in Section 2.1, with a 3 pm spectral resolution and a spatial discretization segment length of Δz = 0.1 mm, providing an optimal balance between resolution and numerical accuracy. Furthermore, active simulations with different grating lengths from 2 to 10 mm, within relevant ranges of the coupling coefficient, pump power, and propagation losses, are analyzed to evaluate the operating regime suitable for monolithic lasers for thermal sensing.

### 3.1. Uniform Heating Sensing

**Figure 1:**
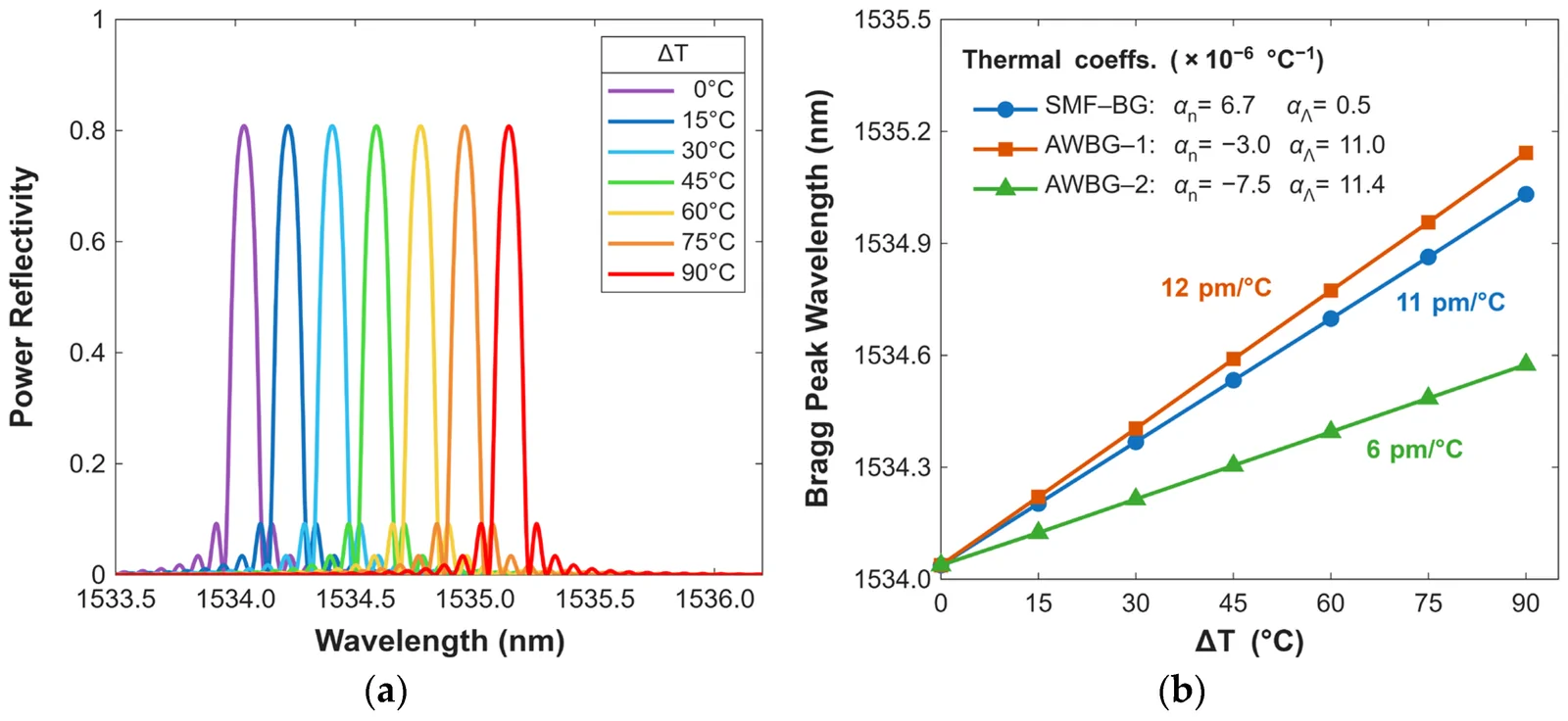
(**a**) Spectral variation and (**b**) linear Bragg peak shift under uniform heating of a 10 mm BG, with 80% reflectivity. Three substrates are compared in (**b**): SMF, single-mode fiber; AWBG–1, our reference phosphate-based sample; AWBG–2, extreme negative thermo-optic coefficient sample.

Figure 1a illustrates the simulated spectral redshift of a 10 mm BG under uniform heating up to 90 °C. A modal overlap factor η= 0.12 is used to simulate an 80% peak reflectivity grating at RT. As derived in Equation (7), the Bragg peak exhibits a linear spectral shift proportional to the sum of the thermo-optic and thermal expansion coefficients. The peak reflectivity remains constant throughout this temperature range: only a 0.1% reduction is observed at ΔT= 100 °C, validating the analytical assumptions regarding the negligible thermal degradation of grating efficiency in this range.

Figure 1b illustrates the Bragg peak shift of three simulated samples, including a reference passive grating inscribed in a standard silica-based single-mode fiber (SMF) [37]. The simulated reference sample (AWBG–1) exhibits a thermal sensitivity $S_{T}=$ 12 pm/°C, calculated via Equation (8) from the Bragg peak drift, which is comparable to that of a simulated passive fiber BG and reported values [11]. However, operating in the AWBG lasing regime provides a distinct advantage: the peak-to-background contrast is several orders of magnitude higher, as shown in Section 3.2.2 and as observed experimentally in our reference AWBG sample. Furthermore, this thermal drift is small enough (shifted up to 1535.3 nm under ΔT= 100 °C) to ensure that the grating continuously operates within the Er^3+^ peak amplification bandwidth, as only minor variations in the emission cross-section occur between 1534 nm and 1535 nm, as checked with some active control simulations centering the resonance at 1535 nm (deviations below 0.5% in the cases closest to the switching transition), validating our assumption on the drift range in Section 2.2. When monitored with a high-resolution optical spectrum analyzer (OSA), as with any passive design based on spectral tracking, this device offers the additional benefit of a narrow, high-contrast emission peak, improving the signal-to-noise ratio compared to equivalent passive designs using the same interrogation setup.

Since the spectral shift sensitivity is intrinsically bound to the material’s thermal coefficients, the choice of the bulk waveguide substrate directly modulates the thermal response. In a phosphate-based glass, its thermal expansion coefficient—higher than that of silica fibers—is partially counterbalanced by its negative thermo-optic coefficient, yielding a similar net sensitivity. Consequently, the composition of the glass matrix could be tailored to reduce the thermal effect, as seen in Figure 1b with sample AWBG–2, which models a phosphate-based glass with a strongly negative thermo-optic coefficient approaching that of the pure phosphate glass (it should be noted that our reference sample stoichiometry is slightly modified in order to stabilize it to certain characteristics). Conversely, although alternative substrates (such as borosilicate-based) could be selected to increase thermal sensitivity, a larger spectral drift would risk shifting the Bragg peak outside the Er^3+^ amplification curve, potentially degrading the laser performance.

### 3.2. Thermal Gradient Sensing

#### 3.2.1. BG Spectral Degradation Under Linear Thermal Gradients

**Figure 2:**
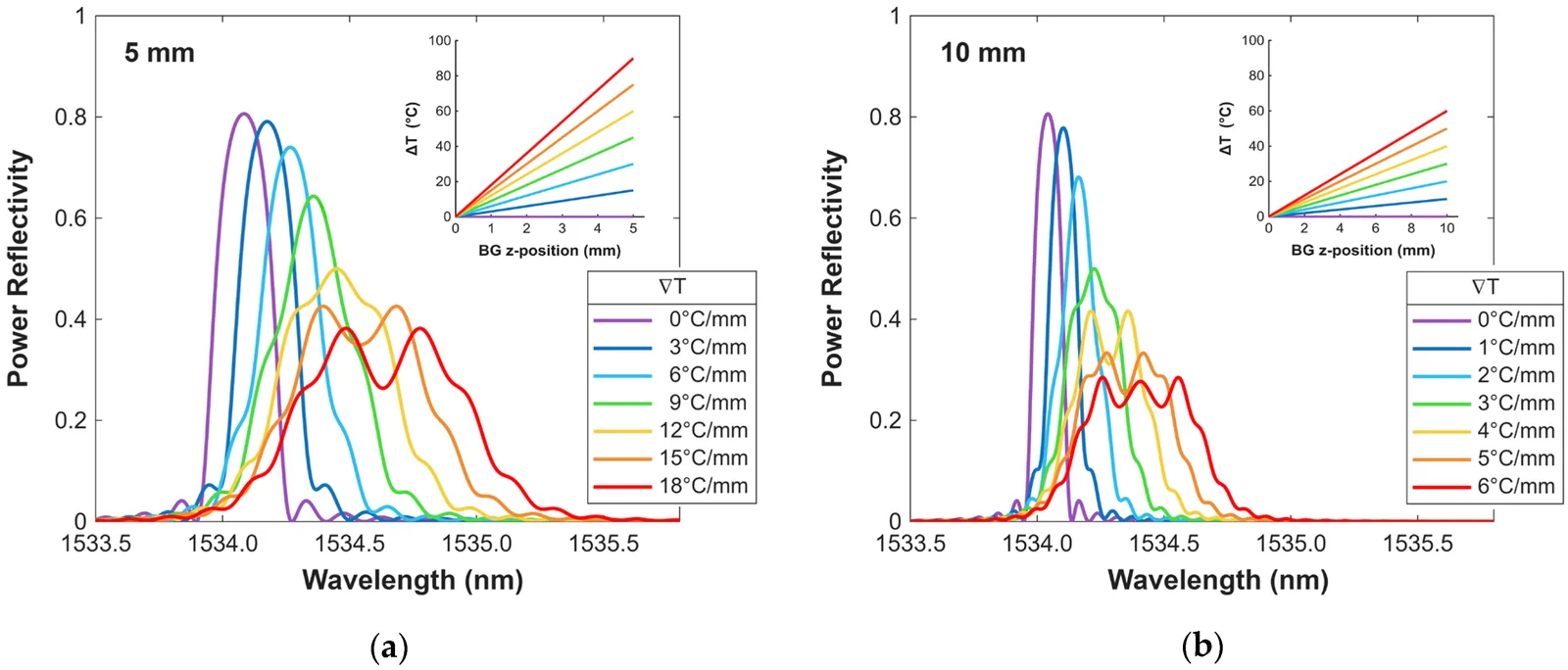
Spectral response under linear thermal gradients of BGs with length (**a**) 5 mm and (**b**) 10 mm. Both gratings are simulated with a baseline reflectivity of 80% under zero-gradient conditions. The insets depict the corresponding linear spatial temperature profile variation with respect to the baseline temperature (RT) applied along the grating axis z due to the thermal gradient.

Figure 2 presents the simulated response of two BGs, with lengths of 5 and 10 mm, under linear longitudinal thermal gradients. Modal overlap factors η= 0.24 and 0.12 are used, respectively, so that an identical baseline peak reflectivity of 80% is simulated in both cases under zero-gradient conditions and RT. To enhance visual clarity and avoid the overlapping of adjacent spectral curves, a Gaussian apodization profile—centered at the grating midpoint and normalized to half of its total length (L/2)—is applied in these specific calculations. This apodization suppresses the characteristic sidelobes caused by Fabry–Perot interference at the grating ends, an effect that becomes more pronounced as the chirp increases, allowing for a clearer observation of the efficiency reduction. The insets in both figures show the spatial linear temperature variation profile from the baseline temperature (z = 0) due to the thermal gradient: $\Delta T\left(z\right)=T\left(z\right)-T\left(0\right)=\nabla T\cdot z$. Gradient ranges up to 18 °C/mm and 6 °C/mm are considered, corresponding to a total difference of 90 °C and 60 °C between the grating ends, respectively. Higher gradients in the 10 mm grating were omitted due to coupling coefficient design constraints, as we will comment on later. In both cases, the BG reflectivity drops significantly from its 80% baseline to a range of only 20–35% depending on the case.

As previously stated, because each point along the grating experiences a different localized temperature, as observed in the inset plots, the spectrum also undergoes a redshift. This shift is equivalent to that induced by the average temperature variation between the grating ends, corresponding to 0.55 nm and 0.37 nm for the 5 and 10 mm gratings, respectively. Furthermore, a longer grating inherently yields a more monochromatic spectral response due to the nature of the cumulative filtering process. However, increasing the grating length also heightens its sensitivity to thermal gradients: the efficiency degradation of the 10 mm grating under 6 °C/mm is much more severe than that of the 5 mm grating under the same gradient, or even under the same end-to-end temperature difference (which corresponds to a 12 °C/mm gradient). This behavior is driven by the cumulative phase detuning within the longer structure. In addition, it should be noted that, as shown in Figure 2, simulated BGs have been carried out with the same baseline reflectivity of 80%: the 5 mm grating requires twice the coupling of the 10 mm case to maintain equal the product κL, and thus an equal baseline reflectivity (Equation (5)). So, the grating response to thermal gradients is highly determined by its physical length and design coupling.

As observed, the induced spectral broadening is remarkably small within our thermal ranges, especially compared to the 1 nm resolution of our active algorithm. This minimal distortion physically justifies the use of the delta-function approximation. Nevertheless, several active control simulations with non-zero transmission coefficients at adjacent wavelengths (modeling small side-lobes) have been conducted (an example is shown and discussed in Section 3.2.2). It is worth noting that the primary application as a thermal gradient detector is through an “on/off” switch of the laser emission, meaning minor variations in the lasing power are of secondary importance as long as the laser remains operational. The main advantage remains the high contrast of the narrow laser line, eliminating the need for costly OSAs. Consequently, the validity of the delta-function approximation adopted in Section 2.2 is again successfully confirmed, even when considering these broadening effects.

#### 3.2.2. Critical Coupling Coefficient and Conditions

If the Bragg grating reflectivity falls below a critical threshold, the self-amplified multiple reflection mechanism is drastically disrupted, quenching the lasing regime [9]. This threshold-governed behavior positions AWBGs as candidates for thermal gradient sensing. The precise critical points are dictated by a combination of structural and operational parameters: grating length, efficiency (coupling coefficient) and the pump power. To analyze the laser performance, the numerical results presented in this section are mapped from the data originally generated in [9]. Specifically, the passive reflectivity values have been translated into the corresponding grating coupling coefficient by means of Equation (5). Since the validity of the delta-function spectral approximation under thermal gradients and the spectral shifts holds, this parameter translation provides an efficient numerical approach avoiding redundant and computationally expensive simulations.

**Figure 3:**
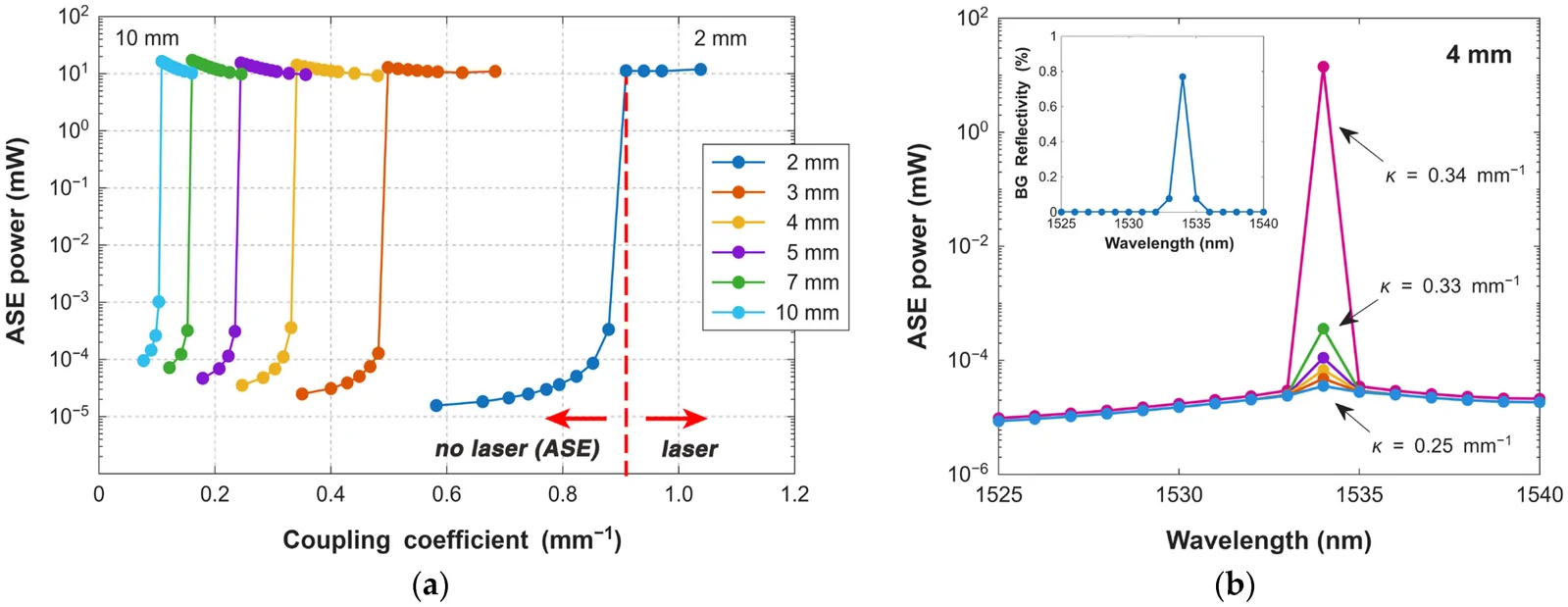
(**a**) ASE power generated by the simulated AWBGs under a pump power of 300 mW as a function of the grating length and its design coupling coefficient. The transition between ASE and lasing regimes in the 2 mm case is marked by a red dashed line. (**b**) Spectral distribution of the ASE output power for the 4 mm grating as a function of the coupling coefficient until the transition to lasing regime is reached (the unlabeled curves correspond to κ= 0.32, 0.30 and 0.28 mm^−1^ from top to bottom). The inset shows the passive BG reflectivity profile considered in the simulations.

Figure 3a presents the ASE output power generated by AWBGs as a function of the length and the design coupling coefficient under a bidirectional 300 mW pump power (this power is launched at each grating end). As observed for each case, the ASE output power initially increases with grating efficiency until reaching a critical coupling threshold where the device enters the lasing regime and the output power jumps by 3–4 orders of magnitude to exceed 10 mW. Figure 3b shows the spectral distribution of the ASE output for the 4 mm case to illustrates the ASE regime prior to the lasing transition. In this case, to refine the BG spectral profile beyond a pure delta function, adjacent wavelengths were assigned a reflectivity equal to 10% of the Bragg reflectivity (inset). Differences observed in these and in other punctual control simulations regarding delta-function specification were not significant (tolerances below 1% in the output power) for the lasing threshold within the coupling sampling resolution employed, confirming that, even under this broader spectral condition, the combination of high amplification occurs exclusively at the Bragg wavelength. This abrupt, threshold-triggered transition offers a peak-to-background contrast drastically higher than that of typical passive devices where the spectral response relies solely on a notch or peak without optical amplification, highlighting the potential of active structures for sensing applications under both this and the uniform heating modes.

To systematically analyze these dependencies, the threshold coupling coefficient ($\kappa_{th}$) is defined in this work as the minimum coupling strength at which the ASE output power reaches saturation and achieves the lasing regime (as noted in the 2 mm curve in Figure 3a). Although the discrete sampling is subject to minor uncertainties, it strikes a balance between numerical accuracy and computational efficiency. Finer resolution across the parameter space would drastically increase computation time, particularly because simulations in the transition region require significantly longer convergence times. As expected, longer gratings require lower $\kappa_{th}$ values to confine the light and achieve lasing due to the larger volume of active material involved.

**Figure 4:**
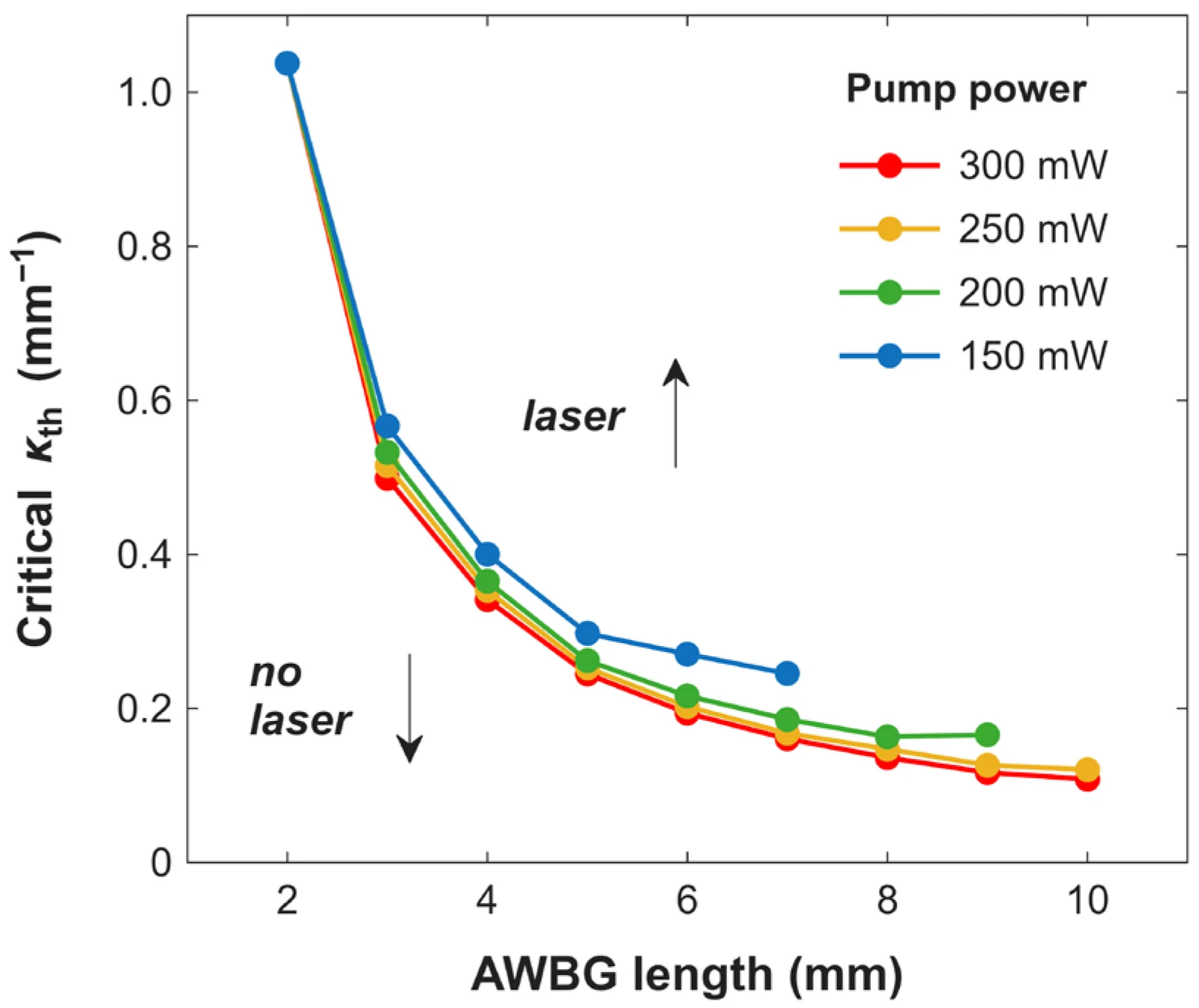
Critical threshold coupling coefficients required to trigger the lasing regime as a function of the AWBG length under four different pump powers.

Figure 4 displays the critical $\kappa_{th}$ values needed to enter the lasing regime as a function of the AWBG length under four different pump powers, obtained from the complete simulation dataset of Figure 3a. Above these threshold curves, the AWBG operates in the lasing regime, whereas below them, the generated output ASE signal is extremely low. Overall, the resulting $\kappa_{th}$ curves show a smooth, continuous trend across the parameter space, confirming that the adopted grid resolution in Figure 3a serves as a reliable approximation despite minor numerical deviations in specific configurations (e.g., the 150 mW pump case for the 5 mm sample). Physically, $\kappa_{th}$ progressively decreases with grating length, as mentioned before, and exhibits a saturation trend. Beyond a certain length, the grating becomes too long for the pump power to sufficiently invert the entire active medium, thereby degrading the device’s performance. On the other hand, lower pump powers require higher critical $\kappa_{th}$ to compensate for the reduced optical gain. This behavior becomes more pronounced for longer gratings, where low pump powers fail to trigger the lasing regime, as observed with the 150 mW pump for lengths above 8 mm, and the 200 mW pump for the 10 mm grating.

#### 3.2.3. Threshold-Triggering Sensor near Critical Conditions

If the AWBG is designed slightly above the critical efficiency required for lasing, any reduction induced by thermal chirp will quench it. To quantify this mechanism, an effective coupling coefficient, $\kappa_{eff}$, is defined as the coupling required to yield the maximum reflectivity of the passive chirped spectrum, accounting for the thermal spectral shift and degradation. Since the analytical coupling expression, Equation (4), does not consider the phase accumulation due to the chirped detuning, $\kappa_{eff}$ is derived numerically by solving Equation (5) using the peak reflectivity extracted from the TMM calculations, translating—as a methodological simplification—this chirped-grating response into a scalar quantity comparable against the uniform-grating threshold dataset. Analyzing the device performance via $\kappa_{eff}$ rather than peak reflectivity provides a direct and more advantageous bridge to its design and fabrication, as two gratings with identical peak reflectivities can exhibit distinct thermal sensitivities (Figure 2).

**Figure 5:**
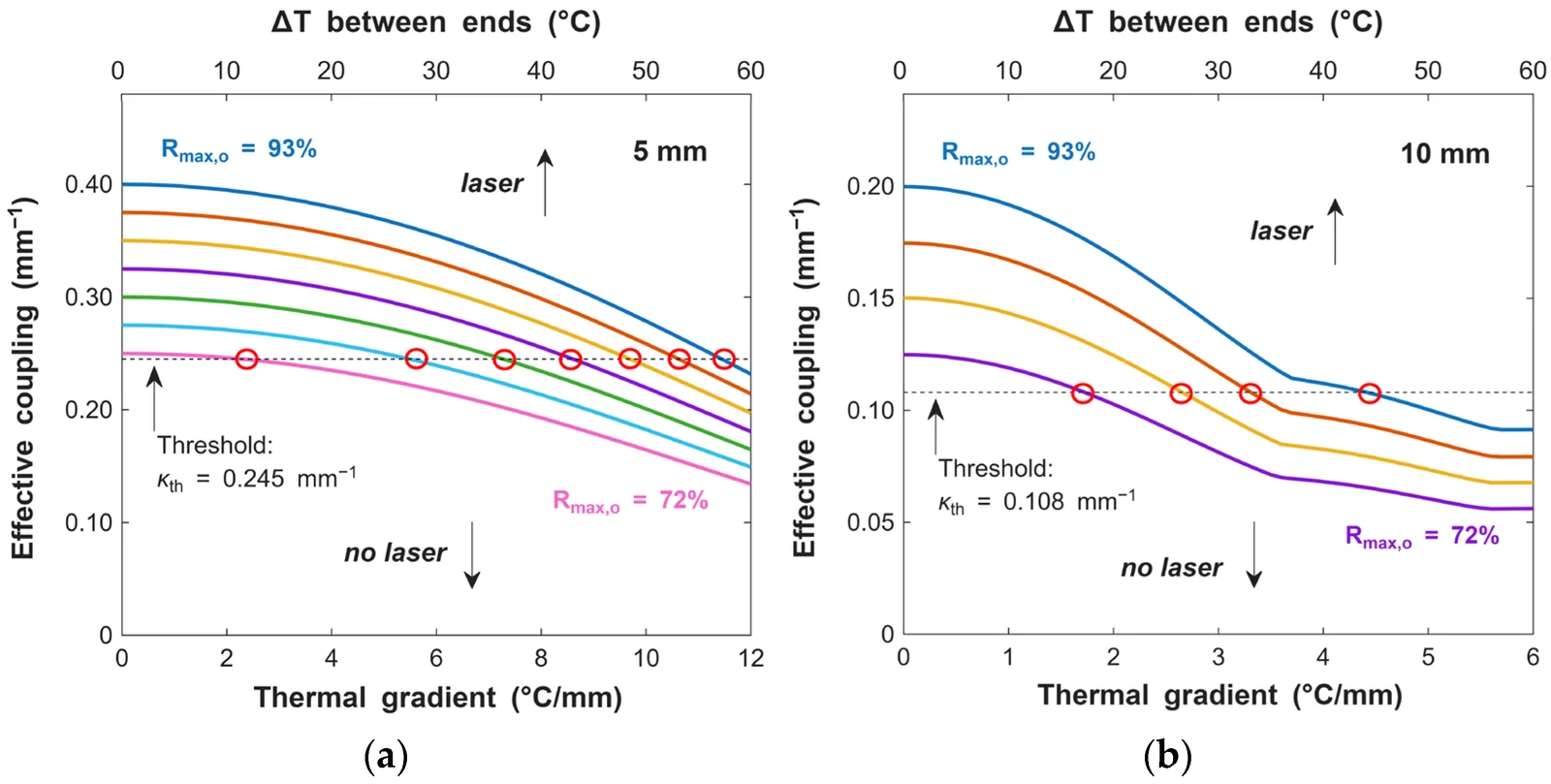
Simulated variation of the effective coupling coefficient as a function of the thermal gradient departing from several design values $\kappa_{o}$ of two AWBGs of length (**a**) 5 mm and (**b**) 10 mm. Both threshold values with 300 mW pump are shown with dashed line. Red circles indicate the thermal gradient at which the grating’s effective coupling reaches the threshold. The total temperature difference between the grating ends is indicated in the upper labels.

Figure 5 illustrates $\kappa_{eff}$ as a function of the thermal gradient for two AWBG lengths, 5 and 10 mm. Simulations incorporate several design coupling values $\kappa_{o}$—that without any thermal gradient—near the lasing threshold, up to a 93% passive reflectivity peak, $R_{o}$, which represents a realistic upper limit for fabrication tolerances. As observed, the coupling decreases as the thermal gradient is increased and, at some gradient value, it falls below the AWBG threshold. The triggering $\kappa_{th}$ for a 300 mW pump is marked with a dashed line. The gradient threshold in which the transition would occur is marked in each curve with a red circle. Specifically, shorter gratings allow for higher-gradient detection, reaching up to 11.5 °C/mm for the 5 mm AWBG, compared to 4.5 °C/mm with the 10 mm grating (both evaluated at the $R_{o}=$ 93% design). This limits the maximum temperature difference across the grating ends to 50–60 °C (top axis), so that the spectral broadening remains negligible (Figure 2) and validates the use of the delta-function profile.

Furthermore, the design coupling window highlights a trade-off between the two grating lengths. The 5 mm design covers a broader coupling range (0.275–0.40 mm^−1^) suited for high gradients, though its sensitivity drops below 6 °C/mm as $\kappa_{eff}$ remains too close to $\kappa_{th}$. Conversely, the 10 mm grating would better resolve lower gradients (1.5–4.5 °C/mm) with well-spaced, achievable design coupling ($\kappa_{o}=$ 0.12–0.20 mm^−1^). This emphasizes the importance of carefully selecting the grating length based on the target longitudinal gradient range, opening promising avenues for combining different geometries in multi-core sensing systems.

**Figure 6:**
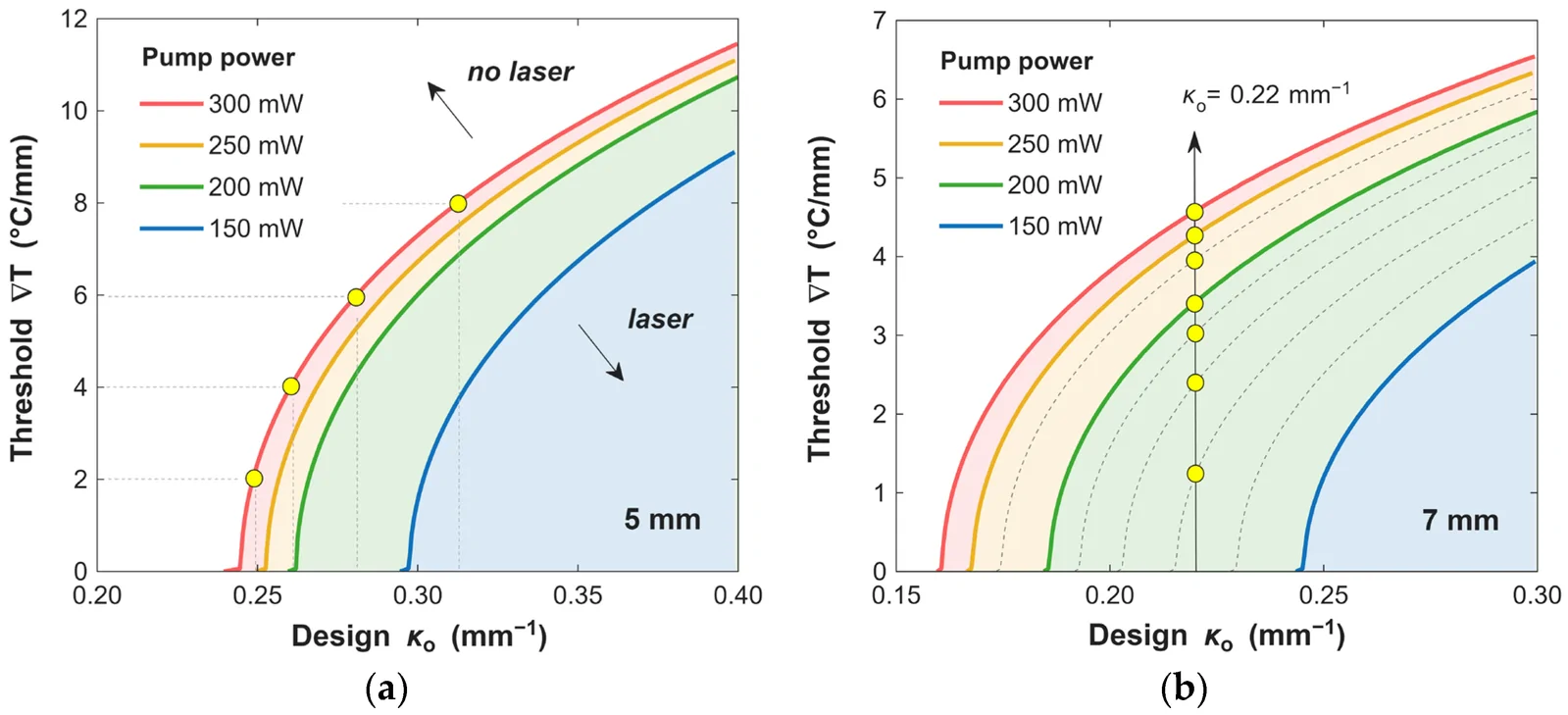
Threshold curves as a function of the design coupling $\kappa_{o}$ for several pump powers. Two AWBG lengths are considered: (**a**) 5 mm and (**b**) 7 mm. In case (**a**), the yellow dots show examples of different triggering thresholds under a 300 mW pump with different fabrication design $\kappa_{o}$. In case (**b**), interpolated curves for intermediate pumps (225, 190, 180, 170 and 160 mW) are shown with dashed lines. Yellow dots indicate pump-tunable triggering threshold for a given $\kappa_{o}$.

The thermal gradient that triggers the AWBG lasing can be obtained for any $\kappa_{o}$ design coupling coefficient. Figure 6 presents the triggering curves for two grating lengths, 5 and 7 mm, under different pump powers. The 7 mm AWBG is shown instead of the 10 mm one to better illustrate the pump dependence. These curves define the boundary gradient beyond which the laser is deactivated for each $\kappa_{o}$ value and pump power. The shaded regions represent the operational regimes where the system maintains lasing under the specific pump power, as labeled in Figure 6a. In Figure 6b, intermediate pump levels are interpolated and depicted with dashed lines. Pump powers beyond 300 mW were omitted from simulations: in longer gratings, the threshold curves begin to exhibit a saturation behavior. This is logical as the rare-earth active behavior reaches gain saturation while the system performance becomes primarily limited by the severe phase distortion within the chirped grating. The longer the grating, the more sensitive the threshold curves to the pump power employed, even failing to trigger the lasing regime in some cases, as shown in Figure 3b.

Two major advantages of the proposed active designs can be highlighted in these results. First, the initial design efficiency of the grating can be deliberately selected to tailor specific detection thresholds, as marked with yellow dots in Figure 6a for a specific pump. Second, for a fixed grating configuration (as that obtained once fabricated), the threshold value of the target thermal gradient can be dynamically adjusted and tuned by simply modulating the applied pump power, as shown with the vertical line in Figure 6b. These capabilities could be exploited in uncoupled-multicore waveguides [37]: if each core is written with a different specific efficiency by adjusting its index modulation depth, the integrated device could provide simultaneous, multi-threshold thermal gradient sensing.

#### 3.2.4. Fabrication Loss Tolerances and Calibration

From a fabrication perspective, femtosecond laser inscription introduces inherent variations, particularly regarding propagation losses, during both core waveguide and index modulation inscriptions. These losses correspond to intrinsic propagation tolerances of the grating itself, rather than to additional non-thermal perturbations. Since the high-sensitivity designs operate close to critical lasing thresholds, such losses can drastically impact performance, consistent with our findings in [9]. Although this introduces the need for calibration of each sample to map the required pump power to each gradient threshold, this limitation can be naturally overcome in the active device framework again via pump regulation.

**Figure 7:**
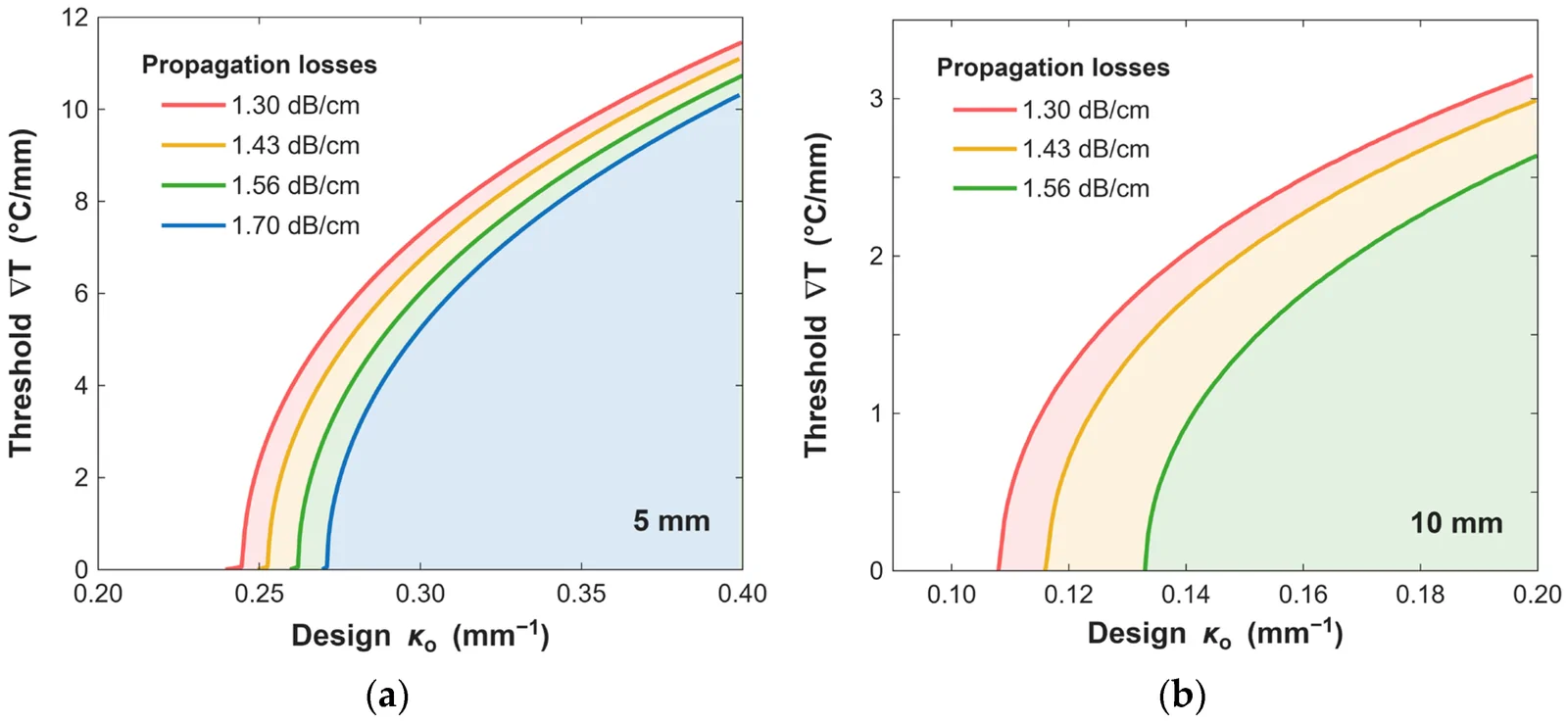
Threshold curves as a function of the $\kappa_{o}$ design coupling with four realistic propagation loss coefficients. Two AWBG lengths are considered under a 300 mW pump: (**a**) 5 mm and (**b**) 10 mm. In the second one, the 1.70 dB/cm loss case does not achieve the lasing regime under this pump power.

Figure 7 presents the threshold gradient curves for two AWBGs of 5 and 10 mm under a 300 mW pump power as a function of the losses. In each case, four realistic propagation loss coefficients after grating fabrication have been considered. As expected, the more losses, the lower the threshold value for the same design. Furthermore, as the grating length increases, the impact of these losses becomes more pronounced, analogous to the pump dependence mentioned before. Above specific losses, the lasing behavior does not occur, as illustrated in Figure 7b, where no lasing region is observed for the 1.70 dB/cm loss case. Thus, once the grating is fabricated, the desired threshold could be calibrated prior to its use by varying the pump. Nevertheless, as observed in Figure 6 regarding saturation with pump power, this calibration might not be feasible if the propagation losses are high or the grating length is more sensitive to them. Therefore, a suitable design has to be adequately chosen beforehand.

It should be noted that direct physical characterization of written structures (e.g., high-resolution refractive index mapping, cross-sectional morphology)—although previously employed in [31,35] to optimize the experimental BG inscription—lies outside the scope of this numerical investigation. Nevertheless, the impact of possible manufacturing tolerances and device-to-device repeatability is inherently addressed through our parametric analysis of propagation losses and coupling strength.

## 4. Conclusions

The numerical framework presented in this work, which couples the TMM with an active-medium propagation algorithm, provides a potential predictive design tool for AWBGs as thermal detectors. Under uniform heating, AWBGs yield thermal sensitivities comparable to passive BG structures, but with the advantage of operating at a high-contrast, narrow laser peak.

The key advantage of the proposed compact AWBG sensor lies in its potential operation as a threshold-triggered thermal gradient detector, for gradients up to approximately 12 °C/mm within the design and thermal ranges considered. This abrupt “on/off” laser switching would allow monitoring using simple, low-cost optical power meters. Unlike equivalent passive sensors that strictly require OSAs to track chirp-induced reflectivity changes, the nature of the AWBG designed close to its lasing threshold simplifies the detection setup. Furthermore, the simulated parameters and operating conditions fall well within feasible fabrication limits, and pump power regulation offers a practical calibration mechanism within a bounded operating window, partially mitigating the technological bottleneck of imperfect control during femtosecond laser inscription. Unlike passive structures whose spectral properties are fixed after fabrication, the AWBG laser threshold can be dynamically adjusted via the pump power, and can also be post-calibrated to compensate for small fabrication loss deviations or to select the detection thresholds within a limited range, provided that an optimal design is chosen beforehand. This capacity makes these designs a promising versatile tunable sensor, a significant novelty compared to conventional BG-based devices limited by their unalterable performance once fabricated.

Although this is a numerical study, the well-established TMM model and the previously validated active algorithm would make these findings transferable to physical device design and optimization prior to fabrication. The results presented, particularly regarding the thermal gradient detector, are obtained under the numerical approximations discussed throughout this work, isolating the thermal-gradient response from other potential cross-sensitivities (e.g., strain, bending, pump fluctuations). They should therefore be regarded as a first quantitative estimate of this device’s potential. Future research could focus on refining some of the approximations and computational resolutions considered here, developing a comprehensive tolerance analysis combining simultaneous variations in the materials, design and operating parameters, and carrying out experimental fabrication and characterization for a definitive validation of the numerical approach. This would also enable a full quantitative, multi-metric comparison against existing sensors (e.g., sensitivity, response time, dynamic range, power consumption). Finally, these findings lay the groundwork for promising directions such as decoupled multi-core architectures for multi-thresholds or transverse gradient sensing, or extending this approach to other physical quantities such as strain, bending or external refractive index detection.

## Figures and Tables

**Table 1:** Simulation parameters of the AWBG, corresponding to our reference experimental sample.

n	$\Delta n_{o}$	DC	$\lambda_{D}$ (nm)	$\alpha_{n}$ (°C^−1^)	$\alpha_{\Lambda }$ (°C^−1^)	β (dB/cm)
1.53095	6 × 10^−3^	5%	1534	−3 × 10^−6^	11 × 10^−6^	1.30

## Data Availability

The original contributions presented in this study are included in the article. Further inquiries can be directed to the corresponding author.
